# Genotype-Dependent Virulence of Severe Fever with Thrombocytopenia Syndrome Virus in a Mouse Challenge Model

**DOI:** 10.3390/ijms27073148

**Published:** 2026-03-30

**Authors:** Eun Bee Choi, Seungyeon Kim, Seo Young Moon, Eun Young Jang, Yookyoung Lee, In-Ohk Ouh

**Affiliations:** Division of Vaccine Development Coordination, Center for Vaccine Research, National Institute of Infectious Diseases, National Institute of Health, Korea Disease Control and Prevention Agency, Osong, Cheongju 28160, Chungcheongbuk-do, Republic of Korea; dmsql2274@korea.kr (E.B.C.); hatmddus135@korea.kr (S.K.); msy1477@korea.kr (S.Y.M.); sky11kk@korea.kr (E.Y.J.); leeykyoung@korea.kr (Y.L.)

**Keywords:** severe fever with thrombocytopenia syndrome virus, genotypes, vaccine

## Abstract

Severe fever with thrombocytopenia syndrome (SFTS) is an emerging tick-borne infectious disease with high case-fatality rates in East Asia. The causative agent, SFTS virus (SFTSV; also known as Dabie bandavirus), exhibits genotype-dependent differences in pathogenicity. However, infection models that recapitulate these variations and can be applied for vaccine and therapeutic evaluation are still lacking. In this study, we assessed the pathogenicity of two Korean SFTSV isolates representing the F and B genotypes in a murine infection model. Wild-type C57BL/6 and IFNAR knockout (IFNAR−/−) mice were intraperitoneally infected with two different doses of SFTSV (2 and 2 × 10^−1^ FFU). All C57BL/6 mice survived regardless of viral genotype or dose. In IFNAR−/− mice, infection with either F- or B-type virus at the 2 FFU dose resulted in mortality beginning at 5 days post-infection, with all mice succumbing within 6 days. At the higher dose (2 × 10^−1^ FFU), mortality differed by genotype: B-type infection led to 20% lethality, whereas F-type infection caused 40% lethality by day 5. Infected and deceased mice exhibited body weight loss as a characteristic clinical outcome. Collectively, these findings demonstrate genotype-associated differences in SFTSV pathogenicity in mice and establish a murine challenge model that may be useful for the preclinical evaluation of candidate vaccines and antiviral agents.

## 1. Introduction

Severe fever with thrombocytopenia syndrome (SFTS) is an emerging infectious disease caused by the SFTS virus (SFTSV), a novel phlebovirus within the family Phenuiviridae. The virus was first identified in rural areas of central China in 2011 and has since been reported in South Korea and Japan [1,2,3]. In addition, another genetically related phlebovirus, Heartland virus (HRTV), has been identified in the United States [4]. The tripartite negative-sense RNA genome of SFTSV consists of the L segment, which encodes the viral RNA-dependent RNA polymerase (RdRp), the M segment encoding the glycoprotein precursors Gn and Gc, and the S segment encoding the nucleocapsid protein (N) and the nonstructural protein (NSs). Clinically, SFTS patients present with high fever, gastrointestinal symptoms, leukocytopenia, and thrombocytopenia [5]. The case fatality rate has been estimated at approximately 20%; however, no specific antiviral therapies or licensed vaccines are currently available for the treatment or prevention of the disease [6,7,8,9]. For this reason, the World Health Organization has designated SFTS as a prioritized pathogen that poses a major threat to public health, highlighting the urgent need for further research and countermeasure development [10].

SFTSV is genetically diverse and has been classified into multiple genotypes (A–F), which exhibit distinct geographic distributions and evolutionary patterns. In South Korea and Japan, genotype B is the predominant circulating strain, whereas genotypes A, D, and F are more frequently reported in China [5,11]. Although genotype F is less frequently reported compared to other genotypes, it has been consistently detected across multiple endemic areas and remains epidemiologically relevant [5]. Previous reports have indicated that genotype B is associated with relatively higher mortality in some cohorts, while genotype F has also been linked to severe disease outcomes despite its lower reported frequency [11,12]. Taken together, these observations suggest that viral prevalence does not necessarily correlate with pathogenic potential and that genotype-specific differences in virulence may exist [11,13].

Animal models that recapitulate human disease are indispensable for elucidating the pathogenesis of SFTSV infection and for the preclinical evaluation of vaccines and therapeutics. However, rhesus macaques, which frequently serve as reliable models for human viral infections, do not develop severe disease following SFTSV challenge and instead exhibit only mild clinical manifestations, including transient fever and hematological abnormalities, without progression to fatal outcomes [14]. Aged ferrets and cats have been shown to exhibit clinical manifestations similar to those of human cases, but they have limited utility in immunological studies compared to murine systems [13,15,16,17]. In immunocompetent laboratory mice, SFTSV infection typically results in mild disease with moderate thrombocytopenia but no mortality. By contrast, mice lacking the type I interferon receptor (IFNAR−/−) exhibit 100% lethality after infection [18,19,20]. Although IFNAR−/− mice lack the initial antiviral defense and therefore have certain limitations, they remain a valuable small animal model for investigating the pathogenesis of severe and fatal SFTS and for testing vaccines and antiviral drugs [21,22,23]. Importantly, IFNAR−/− mice have also been widely applied in studies of other emerging viruses, including Zika virus, Rift Valley fever virus, and chikungunya virus [24,25,26].

In the present study, we investigated the pathogenic differences in SFTSV isolates belonging to the F and B genotypes in IFNAR−/− mice. We selected these two genotypes to represent both a predominant circulating strain (genotype B) and a genetically distinct but clinically relevant genotype (genotype F), enabling a direct comparison of genotype-dependent virulence under controlled host conditions. Based on these findings, we aimed to establish a murine challenge model suitable for evaluating the efficacy of candidate vaccines and therapeutics. Furthermore, we performed pathological examinations of lethal IFNAR−/− infections to assess their histopathological resemblance to fatal human SFTS cases.

## 2. Results

### 2.1. Susceptibility of C57BL/6 and IFNAR−/− Mice to B- and F-Type SFTSV Infection and LD50 Determination

**Figure 1:**
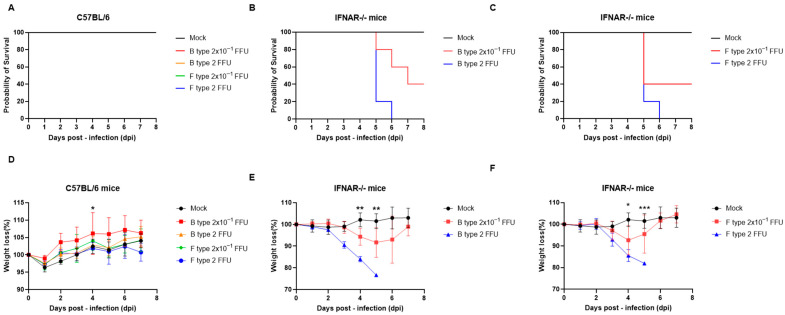
Survival and body weight changes in C57BL/6 and IFNAR−/− mice following inoculation with B- and F-type SFTSV. (**A**–**C**), Mice in each group were intraperitoneally inoculated with the indicated doses of B-type or F-type SFTSV. Survival of C57BL/6 mice infected with B-type and F- type SFTSV (**A**), IFNAR−/− mice infected with B-type SFTSV (**B**), and IFNAR−/− mice infected with F-type SFTSV (**C**) was monitored daily. (**D**–**F**), Relative body weight changes following infection are shown for C57BL/6 mice infected with B-type and F-type SFTSV (**D**), IFNAR−/− mice infected with B-type SFTSV (**E**), and IFNAR−/− mice infected with F-type SFTSV (**F**). Body weight is presented as a percentage of the initial weight at day 0 post-infection. Body weight data were analyzed using a mixed-effects model (REML) with time and group as fixed effects, followed by Šídák’s multiple comparisons test. Data are presented as mean ± s.e.m. Statistical significance is indicated as *p* < 0.05 (*), *p* < 0.01 (**) and *p* < 0.001 (***).

SFTSV isolates of B and F types were intraperitoneally inoculated into WT (C57BL/6) and IFNAR−/− mice at doses of 2 FFU or 2 × 10^−1^ FFU, and survival and body weight changes were monitored. All WT mice survived throughout the observation period, maintaining 100% survival regardless of viral genotype or inoculation dose (Figure 1A). Consistently, body weight in WT mice remained stable or showed a slight increase, indicating minimal clinical impact following infection (Figure 1D).

In contrast, IFNAR−/− mice exhibited marked susceptibility to both genotypes. Following inoculation with the B-type isolate at 2 FFU, mortality began at 5 dpi and reached 100% by 6 dpi. At the lower dose (2 × 10^−1^ FFU), deaths occurred between 5 and 7 dpi, while the remaining mice survived until the end of the observation period (Figure 1B). Similarly, inoculation with the F-type isolate at 2 FFU resulted in complete mortality by 6 dpi, with deaths initiating at 5 dpi. At 2 × 10^−1^ FFU, partial mortality was observed beginning at 5 dpi, while surviving animals remained alive until 8 dpi (Figure 1C). Based on these dose–response survival data, the LD_50_ was calculated as 6.3 × 10^−1^ FFU for the B-type isolate and 6.3 × 10^−2^ FFU for the F-type isolate.

Body weight analysis revealed progressive weight loss in IFNAR−/− mice, particularly in the higher-dose (2 FFU) groups, beginning at approximately 3–5 dpi and temporally coinciding with the onset of mortality (Figure 1E,F). In contrast, mice in the lower-dose groups exhibited more moderate weight loss, with partial recovery observed in surviving animals. Statistical analysis using a mixed-effects model (REML) with Šídák’s multiple comparisons test revealed significant differences at specific time points. In C57BL/6 mice, a significant difference was detected at 4 dpi. In IFNAR−/− mice infected with B-type SFTSV, significant differences were observed at 4 and 5 dpi. In IFNAR−/− mice infected with F-type SFTSV, significant differences between Mock and F-type (2 FFU) groups were observed at 4 dpi (* *p* < 0.05) and 5 dpi (** *p* < 0.0001).

Collectively, these results indicate that WT mice are resistant to lethal disease caused by both SFTSV genotypes, whereas IFNAR−/− mice develop severe disease in a dose-dependent manner characterized by rapid weight loss and high mortality. Notably, the F-type isolate exhibited higher virulence, reaching the LD_50_ at an approximately 10-fold lower inoculum than the B-type isolate.

### 2.2. Hematological Alterations in C57BL/6 and IFNAR−/− Mice Following LD50-Dose Challenge with B- and F-Type SFTSV Isolates

**Figure 2:**
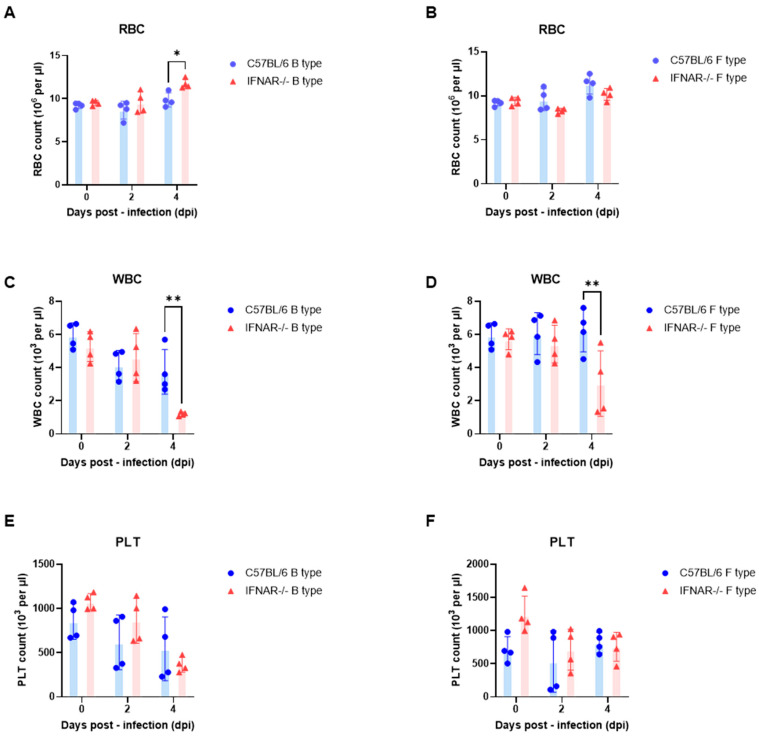
Hematological changes in C57BL/6 and IFNAR−/− mice following LD_50_-dose challenge with B- and F-type SFTSV isolates. (**A**,**B**) Red blood cell (RBC) counts in mice infected with B-type (**A**) or F-type (**B**) SFTSV measured at 0, 2, and 4 days post-infection (dpi). (**C**,**D**) White blood cell (WBC) counts in mice infected with B-type (**C**) or F-type (**D**) SFTSV measured at 0, 2, and 4 dpi. (**E**,**F**) Platelet (PLT) counts in mice infected with B-type (**E**) or F-type (**F**) SFTSV measured at 0, 2, and 4 dpi. C57BL/6 (WT) and IFNAR−/− mice were intraperitoneally inoculated with SFTSV at previously determined LD_50_ doses. Data are presented as mean ± s.e.m., and each point represents an individual animal. Statistical analysis was performed using two-way ANOVA with Šídák’s multiple comparisons test. Significance is indicated as *p* < 0.05 (*), *p* < 0.01 (**).

In contrast, WBC counts showed marked host-dependent alterations. In B-type infection, WT mice exhibited a gradual decline in WBC counts after infection, whereas IFNAR−/− mice displayed a pronounced decrease at 4 dpi (Figure 2C). A similar pattern was observed in F-type infection, where WBC levels remained relatively stable in WT mice but declined markedly in IFNAR−/− mice at 4 dpi (Figure 2D). These results indicate substantial leukocyte reduction in IFNAR−/− mice following infection.

PLT counts decreased after infection in both WT and IFNAR−/− mice under both B-type and F-type infection conditions (Figure 2E,F). Although reductions were detectable from 2 dpi, PLT levels showed notable inter-individual variability, particularly at later time points.

In addition to hematological parameters, serum biochemical markers of tissue injury were evaluated (Supplementary Figure S1). Serum AST and ALT levels tended to increase following infection, with more variable elevations observed in IFNAR−/− mice. F-type infection showed a tendency toward higher enzyme levels compared with B-type infection, although variability among individual animals was noted.

Collectively, these findings indicate that under LD_50_ challenge conditions, RBC levels remain largely stable, whereas WBC and PLT counts exhibit more pronounced alterations following SFTSV infection, particularly in IFNAR−/− mice.

### 2.3. Tissue Viral Loads in C57BL/6 and IFNAR−/− Mice Following LD50-Dose Challenge with B- and F-Type SFTSV Isolates

**Figure 3:**
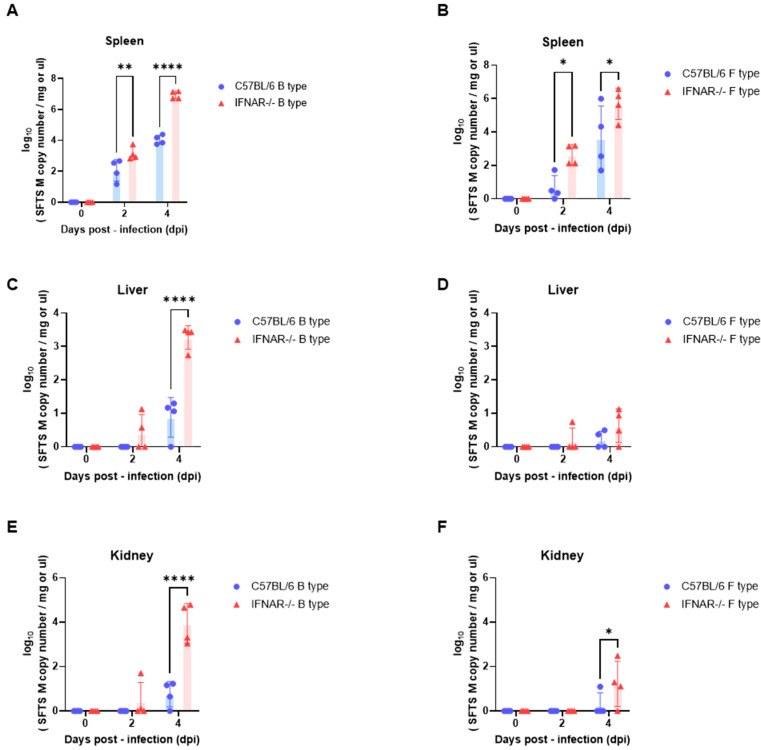
Tissue viral RNA loads in C57BL/6 and IFNAR−/− mice following LD_50_-dose challenge with B- and F-type SFTSV isolates. (**A**,**B**) Viral RNA loads in the spleen following infection with B-type (**A**) and F-type (**B**) SFTSV. (**C**,**D**) Viral RNA loads in the liver following infection with B-type (**C**) and F-type (**D**) SFTSV. (**E**,**F**) Viral RNA loads in the kidney following infection with B-type (**E**) and F-type (**F**) SFTSV. C57BL/6 (WT) and IFNAR−/− mice were intraperitoneally inoculated with SFTSV at previously determined LD_50_ doses. Tissues were collected at 0, 2, and 4 days post-infection (dpi), and viral RNA levels were quantified by qPCR and expressed as SFTSV M gene copy numbers per mg of tissue. Data are presented as mean ± s.e.m., with individual points representing individual animals. Statistical analysis was performed using two-way ANOVA with Šídák’s multiple comparisons test. Significance levels are indicated as *p* < 0.05 (*), *p* < 0.01 (**), and *p* < 0.0001 (****).

WT (C57BL/6) and IFNAR−/− mice were intraperitoneally inoculated with SFTSV at the previously determined LD_50_ doses (B-type: 6.3 × 10^−1^ FFU; F-type: 6.3 × 10^−2^ FFU). Viral loads in the spleen, liver, and kidney were quantified by qPCR at 0, 2, and 4 days post-infection (dpi) and are presented as SFTSV M gene copy numbers (Figure 3).

In the spleen, viral RNA levels increased over time following infection with both B-type and F-type isolates (Figure 3A,B). At 2 dpi, viral RNA was already detectable in IFNAR−/− mice, whereas levels remained low in WT mice. By 4 dpi, IFNAR−/− mice showed markedly higher viral loads compared with WT mice under both genotype infections.

In the liver, viral RNA levels remained close to baseline in WT mice throughout the observation period (Figure 3C,D). In contrast, IFNAR−/− mice exhibited detectable viral RNA levels at 4 dpi following infection with both isolates, with greater variability among individuals.

In the kidney, viral RNA levels were minimal in WT mice across all time points (Figure 3E,F). However, IFNAR−/− mice showed detectable viral RNA at 4 dpi, particularly following infection with the B-type isolate, where higher viral copy numbers were observed.

Overall, these results show that viral RNA levels increase over time in IFNAR−/− mice following LD_50_ challenge and become detectable in multiple organs, whereas viral RNA levels remain low or undetectable in WT mice under the same infection conditions.

### 2.4. Histopathological Changes in the Spleen, Liver, and Kidney of C57BL/6 and IFNAR−/− Mice Following LD50-Dose Challenge with B- and F-Type SFTSV Isolates

**Figure 4:**
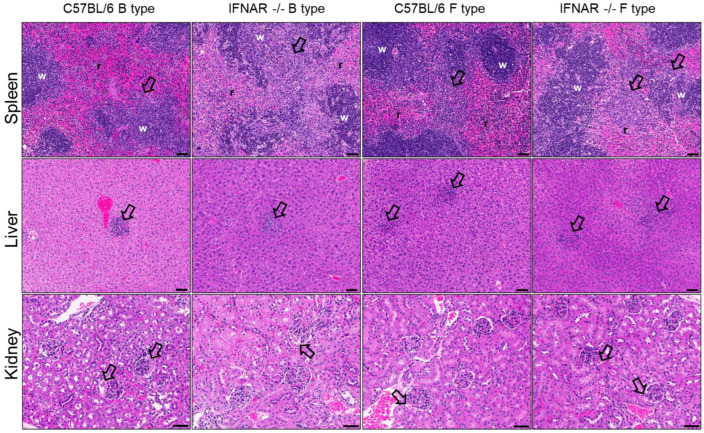
Representative histopathological features of the spleen, liver, and kidney in C57BL/6 and IFNAR−/− mice infected with B- and F-type SFTSV at 4 days post-infection (4 dpi). The top row shows spleen, the middle row liver, and the bottom row kidney tissues. Each tissue was obtained from C57BL/6 (WT) and IFNAR−/− mice infected with B-type or F-type SFTSV. Spleen (**top**): Hyperplasia of reticuloendothelial cells in the marginal zone extending into the red pulp (r) and white pulp (w) (arrows) is observed. Liver (**middle**): Multifocal inflammatory foci scattered within the hepatic parenchyma (arrows) are observed. Kidney (**bottom**): Purplish proteinaceous material within the glomerular (Bowman’s) spaces (arrows) is observed. All sections represent SFTS 4 dpi tissues, stained with hematoxylin and eosin (H&E). Histopathological severity was evaluated using a semi-quantitative scoring system as summarized in Table 1, Table 2 and Table 3. Scale bars = 50 μm.

Pathological findings in the spleen, liver, and kidneys following infection with B- and F-type SFTSV were evaluated (Figure 4). Early histopathological alterations were detectable at 2 dpi (Supplementary Figure S2), indicating initial systemic involvement prior to overt tissue injury observed at later time points. These early changes were generally mild and were characterized by limited reticuloendothelial cell hyperplasia in the spleen, mild hepatic alterations, and minimal renal involvement, with slightly more evident changes in IFNAR−/− mice compared to WT mice (Supplementary Tables S1–S3).

At 4 dpi, more pronounced pathological findings were observed (Figure 4). In the spleen, reticuloendothelial cell proliferation and white pulp atrophy/loss were markedly increased, particularly in IFNAR−/− mice, whereas WT mice exhibited only minimal changes. Additional features, including apoptotic cells within the white pulp, focal inflammation, and pigment deposition, were predominantly observed in IFNAR−/− mice and were consistent with increased immune cell depletion and tissue remodeling (Table 1).

In the liver, oval cell proliferation was generally mild across all groups, with no apparent differences between genotypes or host backgrounds. However, inflammatory foci were more prominent in IFNAR−/− mice, particularly following F-type infection, and were consistent with increased hepatic inflammatory responses (Table 2). Early-stage liver findings at 2 dpi were minimal but included mild hepatocytic changes and limited inflammatory responses (Supplementary Table S2).

In the kidney, proteinaceous material within glomerular spaces was observed across groups and became more apparent at later time points. Although renal changes were generally mild, they were more consistently detected in IFNAR−/− mice (Table 3; Supplementary Table S3).

Collectively, these findings demonstrate that histopathological alterations progress over time and are more pronounced in IFNAR−/− mice, particularly in lymphoid tissues, while certain features such as hepatic inflammation may differ depending on viral genotype.

Table 1 shows histopathological findings in the spleen of C57BL/6 and IFNAR−/− mice following LD_50_-dose challenge with B- and F-type SFTSV at 4 days post-infection (4 dpi). Histopathological features were evaluated in the spleen of C57BL/6 (WT) and IFNAR−/− mice infected with B-type or F-type SFTSV. Lesions were assessed using a semi-quantitative scoring system based on the severity of histological changes.

Table 2 shows histopathological findings in the liver of C57BL/6 and IFNAR−/− mice following LD_50_-dose challenge with B- and F-type SFTSV at 4 days post-infection (4 dpi). Histopathological features were evaluated in the liver of C57BL/6 (WT) and IFNAR−/− mice infected with B-type or F-type SFTSV. Lesions were assessed using a semi-quantitative scoring system based on the severity of histological changes.

Table 3 shows histopathological findings in the kidney of C57BL/6 and IFNAR−/− mice following LD_50_-dose challenge with B- and F-type SFTSV at 4 days post-infection (4 dpi). Histopathological features were evaluated in the kidney of C57BL/6 (WT) and IFNAR−/− mice infected with B-type or F-type SFTSV. Lesions were assessed using a semi-quantitative scoring system based on the severity of histological changes.

## 3. Discussion

SFTSV infection is increasingly recognized as an important public health concern; however, the pathogenic mechanisms underlying disease severity remain incompletely understood, partly due to the limited availability of suitable animal models [1,2,3,4,5,6]. Although IFNAR−/− mice are known to exhibit high susceptibility to SFTSV infection [18,19,20], direct comparisons of virulence among different viral genotypes under identical host conditions remain limited. In this study, we compared the susceptibility of WT and IFNAR−/− mice to B- and F-type SFTSV isolates and integrated survival, hematological, virological, and pathological analyses to evaluate genotype-dependent pathogenicity.

IFNAR−/− mice were used as a susceptible model to investigate genotype-dependent differences in SFTSV pathogenicity, based on the critical role of type I interferon signaling in restricting early viral replication and systemic dissemination. While wild-type mice effectively control infection, IFNAR−/− mice lack this antiviral pathway, leading to uncontrolled viral replication and rapid disease progression [18,19,20]. Mechanistically, impaired induction of interferon-stimulated genes contributes to systemic dissemination and immune dysregulation, including leukopenia and splenic white pulp atrophy, which resemble features of severe human SFTS [11,12]. These findings support the use of the IFNAR−/− mouse model as a sensitive system for evaluating genotype-dependent differences in virulence.

A central observation of this study is the approximately 10-fold lower LD_50_ of the F-type isolate compared with the B-type isolate, indicating that the F-type virus induces lethal disease at lower inoculum doses under IFNAR-deficient conditions. However, this difference was not consistently accompanied by higher viral RNA levels or greater histopathological severity. These results suggest that LD_50_ reflects the efficiency of systemic disease progression rather than viral replication at individual organ sites. Therefore, genotype-dependent virulence likely arises from a complex interplay of tissue tropism, replication kinetics, immune evasion, and host response dynamics [11].

Hematological alterations observed in this study further support this interpretation. While RBC levels remained relatively stable, marked leukopenia and thrombocytopenia were detected in IFNAR−/− mice. Similar hematological abnormalities have been reported in severe human SFTS cases and are considered important clinical indicators associated with disease severity and mortality [11,12]. The splenic reticuloendothelial cell proliferation and white pulp atrophy observed histologically may reflect underlying immune cell depletion and immune dysregulation contributing to these hematological abnormalities.

Analysis of tissue viral RNA loads revealed a characteristic pattern of viral dissemination. Viral amplification was first detected in the spleen and subsequently appeared in peripheral organs including the liver and kidney, particularly in IFNAR−/− mice. Previous animal studies have also identified the spleen as an important early replication site for SFTSV infection [27]. The observed increase in viral RNA levels in the kidney, particularly following B-type infection, may suggest genotype-dependent differences in tissue tropism.

Histopathological findings were broadly consistent with these virological patterns. IF-NAR−/− mice showed prominent splenic alterations including reticuloendothelial proliferation and white pulp atrophy, reflecting lymphoid depletion and impaired immune function. In contrast, hepatic and renal lesions remained relatively mild despite detectable viral RNA levels, suggesting that structural tissue damage may lag behind molecular evidence of infection [11,19].

Consistent with previous studies showing that variations in the viral RdRp can influence replication efficiency and virulence, our findings highlight the importance of genotype-specific characteristics in SFTSV pathogenesis [28]. In particular, the differential pathogenicity observed between genotypes B and F suggests that genetic heterogeneity among circulating strains may contribute to variations in disease severity. Therefore, investigating genotype-dependent pathogenicity is essential for understanding SFTSV biology and supporting the development of effective vaccines and antiviral strategies.

Taken together, these findings suggest that SFTSV virulence is not determined solely by viral replication levels but rather by complex interactions between viral genotype, host immune responses, and systemic disease progression dynamics. Under conditions of impaired type I interferon signaling, these interactions may lead to rapid immune dysregulation, uncontrolled viral dissemination, and lethal disease [20].

In conclusion, this study demonstrates that genotype-dependent differences in SFTSV pathogenicity can be detected in the IFNAR−/− mouse model and that LD_50_ differences may reflect variations in systemic disease progression rather than simple differences in viral replication capacity. These results further support the use of IFNAR−/− mice as a valuable preclinical model for studying SFTS pathogenesis and for evaluating candidate vaccines and antiviral therapies [9,21,22,23]. Establishment of SFTSV infection animal models reflecting currently circulating viral strains may facilitate translational application of these findings to clinical practice.

Several limitations should be acknowledged. The observation period was restricted to early infection stages (0–4 dpi), and viral quantification relied solely on qPCR-based RNA detection, which does not necessarily reflect infectious viral titers. Furthermore, detailed immunological profiling was not performed. Future studies incorporating infectious virus titration, cytokine analysis, immune cell profiling, and immunohistochemical approaches will provide deeper insight into genotype-specific mechanisms of SFTSV pathogenicity.

## 4. Materials and Methods

### 4.1. Virus Strains and Propagation

Korean SFTSV isolates representing the B genotype (GenBank accession nos. KP663743–KP663745) and the F genotype (GenBank accession nos. KF358691–KF358693) were kindly provided by Professor Nam-Hyuk Cho. Viruses were propagated in monolayers of Vero E6 cells (ATCC no. CRL-1586; American Type Culture Collection) maintained in Minimum Essential Medium (MEM; Gibco, Grand Island, NY, USA) supplemented with 2% fetal bovine serum (Gibco) and antibiotics (penicillin, 100 U/mL; streptomycin, 100 μg/mL; Gibco) at 37 °C in an incubator with 5% CO_2_. Cell culture supernatant was collected at 5 dpi and stored at −80 °C as the working virus stock for animal studies. Viral infectivity titres were determined by a focus-forming assay (FFA) using Vero E6 cells, and titres were expressed as focus-forming units per millilitre (FFU/mL).

### 4.2. Animal Infection and Sample Collection

Female C57BL/6 wild-type (WT) mice were purchased from DBL Co., Ltd. (Eumseong, Republic of Korea), and age-matched type I interferon receptor-deficient (IFNAR−/−) mice (B6.129S2-Ifnar1<tm1Agt>/Mmjax, stock no. 028288) were obtained from The Jackson Laboratory. Mice aged 13–15 weeks were used to establish the infection model.

To determine the median lethal dose (LD_50_), mice were intraperitoneally (i.p.) inoculated with B-type or F-type SFTSV at doses of 2 FFU or 2 × 10^−1^ FFU in 100 μL of phosphate-buffered saline (PBS). Mice were assigned to experimental groups according to viral genotype and inoculation dose, with four animals per group (*n* = 4 per group).

Survival data were used to calculate LD_50_ values using the Reed–Muench method. The LD_50_ was determined to be 6.3 × 10^−1^ FFU for the B-type isolate and 6.3 × 10^−2^ FFU for the F-type isolate. These LD_50_ doses were subsequently used for all downstream experiments, including hematological analysis, tissue viral RNA quantification, and histopathological evaluation.

For sample collection, blood and tissue samples (liver, spleen, and kidney) were obtained at 2 and 4 days post-infection (dpi). Blood samples were collected from the retro-orbital venous plexus and analyzed using an automated hematology analyzer (Exigo-Vet, Boule Medical AB Inc., Stockholm, Sweden). Serum samples were separated and stored at −80 °C until further analysis.

At the end of the experiments, infected animals were humanely euthanized and major organs were aseptically harvested for further analysis.

All SFTSV infection experiments were conducted in an animal biosafety level 3 (BSL-3) facility.

Animal procedures were reviewed and approved by the Institutional Animal Care and Use Committee (IACUC) of the Korea Disease Control and Prevention Agency (approval number: KDCA-IACUC-24-056).

### 4.3. RNA Extraction and Quantitative Real-Time PCR

Total RNA was extracted from serum and tissue homogenates using the RNeasy Mini Kit (QIAGEN, Hilden, Germany) according to the manufacturer’s instructions. Detection and quantification of SFTSV RNA were performed using a commercial SFTSV real-time RT-PCR kit (IR5010SD, Kogene Biotech, Seoul, Republic of Korea) on a QuantStudio™ 5 Real-Time PCR System (Thermo Fisher Scientific, Waltham, MA, USA), following the manufacturer’s protocol. Ct values were determined automatically by the instrument software, and samples were considered positive when Ct values were ≤40.

### 4.4. Histopathology

In the SFTSV infection study, four mice per group were necropsied at 2 and 4 days post-infection (dpi). Liver, spleen, and kidney tissues were collected for histopathological examination. Tissues were fixed in 4% formalin and processed for paraffin embedding according to standard procedures. Paraffin-embedded tissues were sectioned at a thickness of 5 μm using a microtome (HM-340E; Thermo Fisher Scientific Inc., Waltham, MA, USA) and mounted on glass slides. Tissue sections were stained with hematoxylin and eosin (H&E) following a standard protocol and examined under a light microscope.

### 4.5. Statistical Analysis

Unless otherwise stated, all statistical analyses were performed using GraphPad Prism software (version 9.0; GraphPad Software, San Diego, CA, USA). Differences between two groups were analyzed using the two-tailed Mann–Whitney U test. For experiments involving two independent variables (genotype and time post-infection), statistical significance was determined using ordinary two-way analysis of variance (ANOVA) followed by Šídák’s multiple comparisons test. A *p* value of <0.05 was considered statistically significant.

## 5. Conclusions

This study provides a systematic evaluation of genotype-dependent virulence of SFTSV using C57BL/6 (WT) and IFNAR−/− mouse models under identical infection conditions. IFNAR deficiency markedly increased susceptibility to infection, as evidenced by dose-dependent mortality, rapid weight loss, hematological abnormalities, increased tissue viral RNA loads, and characteristic splenic pathological alterations. These findings highlight the critical role of type I interferon signaling in restricting early viral replication and preventing systemic dissemination.

Genotype-specific differences in pathogenicity were also identified. The F-type isolate exhibited an approximately 10-fold lower LD_50_ than the B-type isolate, indicating that the F-type virus reaches the lethal threshold at substantially lower inoculum doses under IFNAR-deficient conditions. Notably, these LD_50_ differences were not consistently associated with higher viral RNA loads or more severe histopathological changes, suggesting that genotype-dependent virulence reflects differences in the ability of viral strains to drive systemic disease progression rather than simply differences in viral replication levels.

Overall, these findings demonstrate that SFTSV pathogenicity is shaped by complex interactions between viral genotype and host immune responses. The IFNAR−/− mouse model therefore represents a robust and sensitive platform for studying genotype-dependent virulence of SFTSV and provides a valuable preclinical system for investigating disease mechanisms and evaluating candidate vaccines and antiviral therapeutics.

## Figures and Tables

**Table 1:** Histopathological findings in the spleen.

Group	IFNAR_B	IFNAR_F	C57BL/6_B	C57BL/6_F
Day	4 dpi	4 dpi	4 dpi	4 dpi
Reticuloendothelial cell hyperplasia, red & white pulp	4+	2+	1+	1+
Loss (atrophy) of white pulp	4+	3+	1+	1+
Apoptotic cells, white pulp	2+	-	-	-
Inflammation (lymphadenitis), focal	-	P	-	-
Pigments, white pulp	2+	-	-	
Pigments, red pulp	1+	1+	-	1+

Criteria for evaluation were defined as follows: 1+ (minimal), 2+ (mild), 3+ (moderate) and 4+ (severe); P indicates presence.

**Table 2:** Histopathological findings in the liver.

Group	IFNAR_B	IFNAR_F	C57BL/6_B	C57BL/6_F
Day	4 dpi	4 dpi	4 dpi	4 dpi
Oval cell proliferation, diffuse	1+	1+	1+	1+
Inflammatory foci with necrotic hepatocytes	1+	3+	1+	1+
No. of inflammatory foci	7	46	4	11
Necrosis and inflammation, a lobe, sub-massive	-	-	-	-
Hepatocytic vacuolation, diffuse	-	-	1+	1+
Hemorrhagic cyst	-	P	-	-

Criteria for evaluation were defined as follows: 1+ (minimal), and 3+ (moderate); P indicates presence.

**Table 3:** Histopathological findings in the kidney.

Group	IFNAR_B	IFNAR_F	C57BL/6_B	C57BL/6_F
Day	4 dpi	4 dpi	4 dpi	4 dpi
Proteinous cast, glomerular spaces	2+	2+	2+	1+
Peritonitis		P		

Criteria for evaluation were defined as follows: 1+ (minimal) and 2+ (mild); P indicates presence.

## Data Availability

All data used in the analyses are provided within this article and its Supplementary Materials. For any additional questions, please reach out to the authors responsible for correspondence.

## Supplementary Materials

The following supporting information can be downloaded at: https://www.mdpi.com/article/10.3390/ijms27073148/s1.
