# Supplementary material for: Genotype-Dependent Virulence of Severe Fever with Thrombocytopenia Syndrome Virus in a Mouse Challenge Model
Source: Int J Mol Sci. 2026 Mar 30;27(7):3148. doi: 10.3390/ijms27073148 (PMC13073972; doi:10.3390/ijms27073148)
Supplement: Supplementary file 1 [file ijms-27-03148-s001.zip › Supplementary Figure S1.pdf]

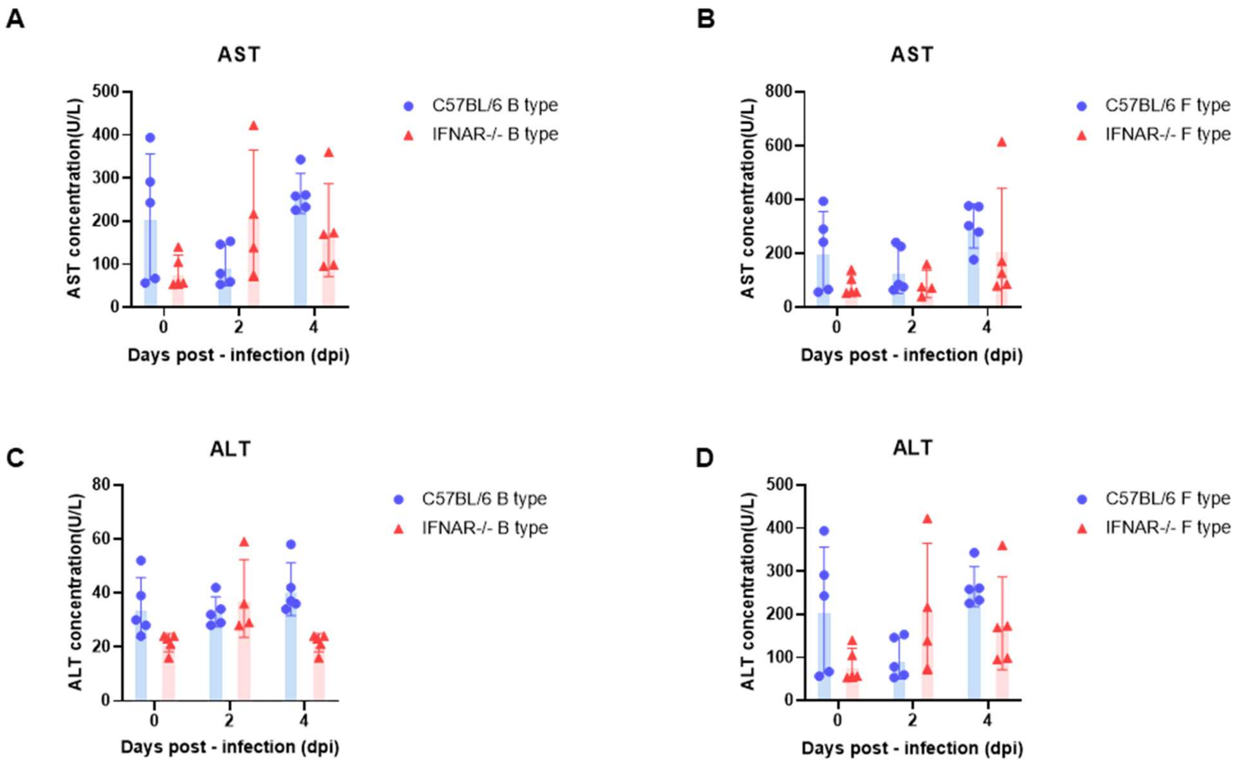

Supplementary Figure S1. Serum biochemical changes in C57BL/6 and IFNAR−/− mice intraperitoneally inoculated with LD_{50} doses of B- and F-type SFTSV isolates. Serum aspartate aminotransferase (AST) levels following infection with B-type (a) and F-type (b) SFTSV. AST concentrations were measured at 0, 2, and 4 days post-infection (dpi). Serum alanine aminotransferase (ALT) levels following infection with B-type (c) and F-type (d) SFTSV, showing time-dependent changes in liver enzyme levels. All data were obtained from C57BL/6 (WT) and IFNAR−/− mice inoculated with previously determined LD_{50} doses. Values are presented as mean ± s.e.m.; dpi, days post-infection; AST, aspartate aminotransferase; ALT, alanine aminotransferase.
