# Supplementary material for: Genotype-Dependent Virulence of Severe Fever with Thrombocytopenia Syndrome Virus in a Mouse Challenge Model
Source: Int J Mol Sci. 2026 Mar 30;27(7):3148. doi: 10.3390/ijms27073148 (PMC13073972; doi:10.3390/ijms27073148)
Supplement: Supplementary file 1 [file ijms-27-03148-s001.zip › Supplementary Figure S2.pdf]

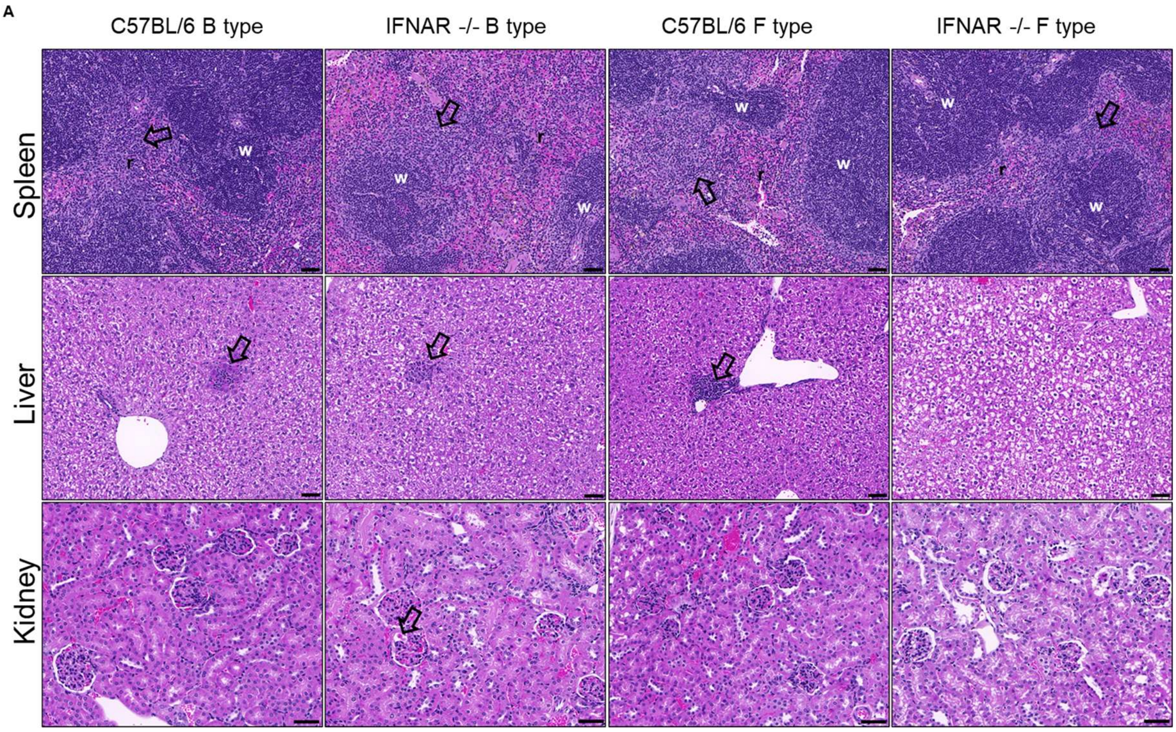

Supplementary Figure S2. Representative histopathological features of the spleen, liver, and kidney in C57BL/6 and IFNAR−/− mice infected with B- and F-type SFTSV at 2 days post-infection (2 dpi). The top row shows spleen, the middle row liver, and the bottom row kidney tissues. Each tissue was obtained from C57BL/6 (WT) and IFNAR−/− mice infected with B-type or F-type SFTSV. Spleen (top): Hyperplasia of reticuloendothelial cells in the marginal zone extending into the red pulp (r) and white pulp (w) (arrows) is observed. Liver (middle): Multifocal inflammatory foci scattered within the hepatic parenchyma (arrows) are observed. Kidney (bottom): Purplish proteinaceous material within the glomerular (Bowman's) spaces (arrows) is observed. All sections represent SFTS 2 dpi tissues, stained with hematoxylin and eosin (H&E). Scale bars = 50 μm.
