# Supplementary material for: Genotype-Dependent Virulence of Severe Fever with Thrombocytopenia Syndrome Virus in a Mouse Challenge Model
Source: Int J Mol Sci. 2026 Mar 30;27(7):3148. doi: 10.3390/ijms27073148 (PMC13073972; doi:10.3390/ijms27073148)
Supplement: Supplementary file 1 [file ijms-27-03148-s001.zip › Supplementary Table S1.pdf]

Supplementary Table S1. Histopathological findings in the spleen of C57BL/6 and IFNAR−/− mice following LD_{50}-dose challenge with B- and F-type SFTSV at 2 days post-infection (2 dpi). Histopathological features were evaluated in the spleen of C57BL/6 (WT) and IFNAR−/− mice infected with B-type or F-type SFTSV. Lesions were assessed using a semi-quantitative scoring system based on the severity of histological changes.

Group	IFNAR_B	IFNAR_F	C57BL/6_B	C57BL/6_F
Day	2dpi	2dpi	2dpi	2dpi
Reticuloendothelial cell hyperplasia, red & white pulp	1+	1+	1+	1+
Loss (atrophy) of white pulp	2+	1+	1+	1+
Apoptotic cells, white pulp				1+
Inflammation (lymphadenitis), focal				
Pigments, white pulp	1+	2+		
Pigments, red pulp	1+	2+		

Criteria for evaluation: 1+, minimal; 2+, mild; 3+, moderate
P, present
