# Supplementary material for: Genotype-Dependent Virulence of Severe Fever with Thrombocytopenia Syndrome Virus in a Mouse Challenge Model
Source: Int J Mol Sci. 2026 Mar 30;27(7):3148. doi: 10.3390/ijms27073148 (PMC13073972; doi:10.3390/ijms27073148)
Supplement: Supplementary file 1 [file ijms-27-03148-s001.zip › Supplementary Table S2.pdf]

Supplementary Table S2. Histopathological findings in the liver of C57BL/6 and IFNAR−/− mice following LD_{50}-dose challenge with B- and F-type SFTSV at 2 days post-infection (2 dpi). Histopathological features were evaluated in the liver of C57BL/6 (WT) and IFNAR−/− mice infected with B-type or F-type SFTSV. Lesions were assessed using a semi-quantitative scoring system based on the severity of histological changes.

Group	IFNAR_B	IFNAR_F	C57BL/6_B	C57BL/6_F
Day	2dpi	2dpi	2dpi	2dpi
Oval cell proliferation, diffuse	1+	1+	1+	1+
Inflammatory foci with necrotic hepatocytes			1+	1+
No. of inflammatory foci#	2	0	2	5
Necrosis and inflammation, a lobe, sub-massive				
Hepatocytic vacuolation, diffuse	2+	4+	1+	2+
Hemorrhagic cyst				

Criteria for evaluation: 1+, minimal; 2+, mild; 3+, moderate; 4, severe
P, present
