# Supplementary material for: Genotype-Dependent Virulence of Severe Fever with Thrombocytopenia Syndrome Virus in a Mouse Challenge Model
Source: Int J Mol Sci. 2026 Mar 30;27(7):3148. doi: 10.3390/ijms27073148 (PMC13073972; doi:10.3390/ijms27073148)
Supplement: Supplementary file 1 [file ijms-27-03148-s001.zip › Supplementary Table S3.pdf]

Supplementary Table S3. Histopathological findings in the kidney of C57BL/6 and IFNAR−/− mice following LD_{50}-dose challenge with B- and F-type SFTSV at 2 days post-infection (2 dpi). Histo-pathological features were evaluated in the kidney of C57BL/6 (WT) and IFNAR−/− mice infected with B-type or F-type SFTSV. Lesions were assessed using a semi-quantitative scoring system based on the severity of histological changes.

**Group**	**IFNAR_B**	**IFNAR_F**	**C57BL/6_B**	**C57BL/6_F**
**Day**	**2dpi**	**2dpi**	**2dpi**	**2dpi**
Proteinous cast, glomerular spaces	1+			
Peritonitis				

Criteria for evaluation: 1+, minimal; 2+, mild

P, present
